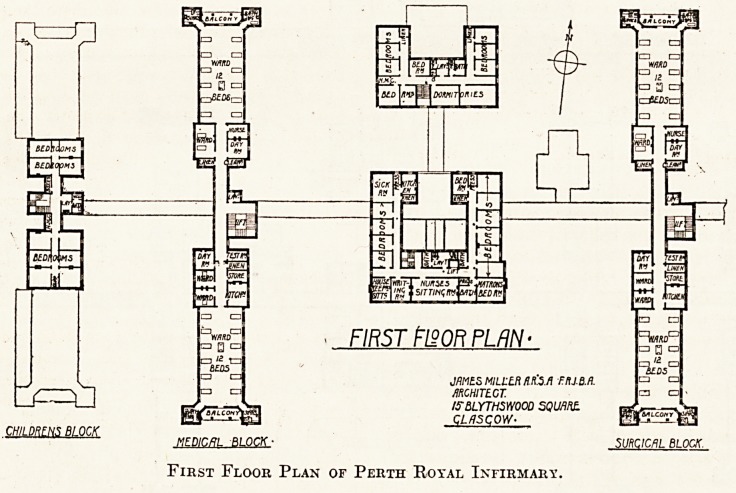# Perth Royal Infirmary

**Published:** 1915-03-13

**Authors:** 


### HOSPITAL ARCHITECTURE AND CONSTRUCTION.

### Perth Royal Infirmary.

This new building has been erected on a site of about 11 acres in extent from designs by Mr. James Miller, A.R.S.A., F.R.I.B.A.

The plan generally is laid out on the lines of the Derbyshire Royal Infirmary and has the administration block in the centre, with a corridor running east and west, and the ward pavilions north and south on each side. There are four pavilions for adult patients and one for children.

The administration block contains the usual offices, quarters for staff, stores, etc., on the ground floor, and on the upper floors bedrooms and sitting rooms for nurses.

The kitchen block is placed at some little distance to the north of the administration block and contains on the ground floor the kitchen offices and stores and servants' mess-room. The kitchen and scullery are lighted from the top, and are therefore one storey in height only. The rest of the block is carried up two additional storeys and provides sleeping accommodation for the servants.

The ward blocks for adult patients on the south side of the corridor each contain a ward for twelve beds, two single-bed wards, ward kitchen, store, linen room, testing room, and day room. At the further end of the large ward are two projecting wings, one of which contains a bathroom, the other a w.c. and sink room.

No provision is made for a nurses' w.c., and, as there is no sanitary accommodation at the other end of the block, vessels used in the two small wards would have to be carried through the large ward to be emptied and cleansed.

The ward blocks on the north side of the corridor are slightly different. At the entrance end of the ward a nurses' room, day room, and cleaner's cupboard occupy one side and a linen room and a ward for two beds occupy the other side. The ward kitchen apparently has to serve for both wards. Looking at the distance between the two wards this seems an impracticable arrangement.

New Buildings of Perth Royal Infirmary.

The staircases are attached to the corridor, on to which they and the lifts open, and a cross-ventilated lobby separates the ward block from the main corridor.

The children's ward blocks are planned much in the same way, except that there is a larger day-room and two nurses' rooms are provided.

All the wards have balconies at the end, which might, we think, with advantage have been made much deeper. To get a bed out and leave sufficient room to pass at least 9 feet is required. These balconies do not seem to be more than about 6 feet 6 inches.

The operation block is placed between the administration block and the east ward block. It contains, besides the theatre, an anæsthetic room, dressings store, sterilising room, and recovery room. As the anæsthetic room does not communicate with the theatre a patient must be wheeled out into the corridor again to get to the theatre; not a very desirable arrangement. There is no wash-up room, so presumably all the sinks and washing apparatus are in the theatre, and there is no surgeon's room.

laundry staff. Attached to the boiler-house is an incinerator for refuse.

To the west of the boiler-house block is a small building containing two wards of two beds each for isolating doubtful or infectious cases, with a nurses' room and kitchen.

At the main entrance is a porter's lodge.

First Floor Plan of Perth Royal Infirmary.

On the other hand, a recovery room is provided: an arrangement that has long been discarded in a modern hospital.

The admission block, which apparently also serves for the treatment of casualties, is at the east end of the main corridor. It contains a waiting room, medical consulting room, operation room, with dressings store and recovery room, and two large rooms for electrical and $x$-ray work.

The entrance and exit for patients are entirely disconnected from the hospital corridor, and the dispensary is so placed that it serves for both out-patients and for the service of the hospital.

The pathological block is a detached building and contains mortuary, *post-mortem* room, laboratory, and waiting room.

To the west of this is the laundry and boiler-house block. This is arranged generally on the usual lines of a modern laundry, and includes a disinfecting room; also a dining room for the

## Figures and Tables

**Figure f1:**
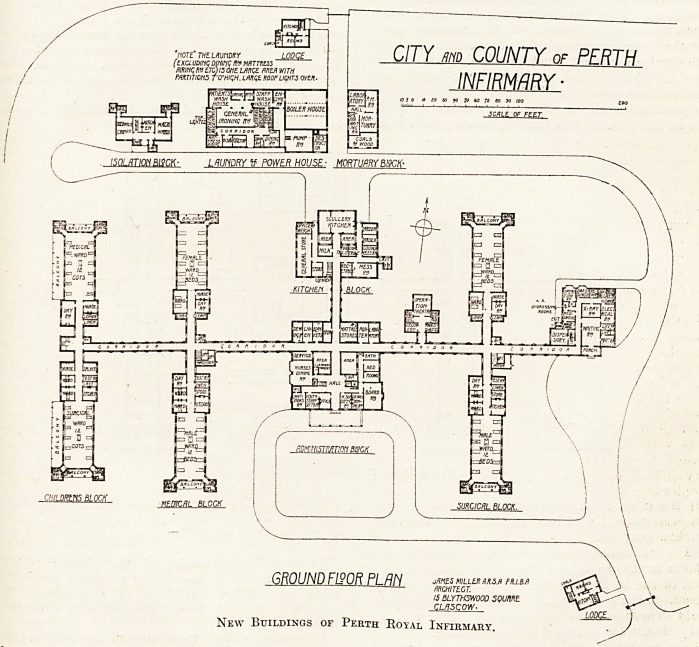


**Figure f2:**